# Attendance at remote versus in-person outpatient appointments in an NHS Trust

**DOI:** 10.1177/1357633X231216501

**Published:** 2023-12-21

**Authors:** Gabriele Kerr, Geva Greenfield, Benedict Hayhoe, Fiona Gaughran, Kristoffer Halvorsrud, Mariana Pinto da Costa, Nirandeep Rehill, Rosalind Raine, Azeem Majeed, Ceire Costelloe, Ana Luisa Neves, Thomas Beaney

**Affiliations:** 1 156434 Department of Primary Care and Public Health, 4615 Imperial College London, London, UK; 2 NIHR Applied Research Collaboration Northwest London, London, UK; 3 Institute of Psychiatry, Psychology and Neuroscience, 34426 King's College London, London, UK; 4 4958 South London and Maudsley NHS Foundation Trust, London, UK; 5 NIHR Applied Research Collaboration South London, London, UK; 6 NIHR Applied Research Collaboration North Thames, London, UK; 7 Department of Applied Health Research, 4919 University College London (UCL), London, UK; 8 117534 Institute of Cancer Research, Sutton, UK

**Keywords:** eHealth, remote consultation, telecare, telehealth, telemedicine

## Abstract

**Introduction:**

With the growing use of remote appointments within the National Health Service, there is a need to understand potential barriers of access to care for some patients. In this observational study, we examined missed appointments rates, comparing remote and in-person appointments among different patient groups.

**Methods:**

We analysed adult outpatient appointments at Imperial College Healthcare NHS Trust in Northwest London in 2021. Rates of missed appointments per patient were compared between remote versus in-person appointments using negative binomial regression models. Models were stratified by appointment type (first or a follow-up).

**Results:**

There were 874,659 outpatient appointments for 189,882 patients, 29.5% of whom missed at least one appointment. Missed rates were 12.5% for remote first appointments and 9.2% for in-person first appointments. Remote and in-person follow-up appointments were missed at similar rates (10.4% and 10.7%, respectively). For remote and in-person appointments, younger patients, residents of more deprived areas, and patients of Black, Mixed and ‘other’ ethnicities missed more appointments. Male patients missed more in-person appointments, particularly at younger ages, but gender differences were minimal for remote appointments. Patients with long-term conditions (LTCs) missed more first appointments, whether in-person or remote. In follow-up appointments, patients with LTCs missed more in-person appointments but fewer remote appointments.

**Discussion:**

Remote first appointments were missed more often than in-person first appointments, follow-up appointments had similar attendance rates for both modalities. Sociodemographic differences in outpatient appointment attendance were largely similar between in-person and remote appointments, indicating no widening of inequalities in attendance due to appointment modality.

## Introduction

Missed appointments result in delays in care, inefficient resource use and worse health outcomes,^1–3^ particularly in patients with poorer health and more complex social needs.^4–6^ The association between missed appointments and health inequalities is well established.^2,7^ In the context of continued significant pressure on the National Health Service (NHS), healthcare providers are increasingly looking toward alternative models of care with the aim of improving efficiency and access to meet demand.^8^ Remote consultations could answer some of these needs because of their increased time efficiency^9,10^ while maintaining standards of care,^11,12^ but evidence for impacts of remote consultations on attendance rates is limited. COVID-19 triggered a rapid shift towards the provision of healthcare remotely, with the intention of safeguarding patients and healthcare staff from risk of infection.^13–15^ This has enabled exploration of the variation in attendance rates by appointment modality, as well as associated patient characteristics.

Remote secondary care services have similar or improved attendance compared to in-person consulting for some patients,^16–23^ potentially attributable to a reduced need to travel or interruption to work and social lives^9,10^ or reduced wait times.^12^ These benefits may mitigate barriers to accessing appointments in individuals restricted by work commitments, travel ability,^24,25^ or at greater risk of severe disease from COVID-19.^14^ Evidence from the United States suggests remote appointments may reduce disparities in non-attendance rates between socioeconomic groups^23^ and between patients with and without chronic health conditions.^26^

However, the increasing use of remote consultations may pose a new barrier to accessing outpatient services, potentially contributing to the ‘digital exclusion’ of vulnerable patient groups and entrenching existing health inequalities.^27,28^ Older age groups, patients without English as a first language, male patients, people from ethnic minority or lower income backgrounds are less likely to be offered or to take up a remote outpatient appointment in secondary or tertiary care.^12,27,29^ It is unclear how much of this demographic variation is due to barriers to accessing remote services or differences in healthcare needs. Additionally, remote modalities may be inappropriate in some clinical circumstances.^13^ For example, audio-only appointment modes do not enable detection of non-verbal cues or clinical signs^30^ and may inhibit establishment of rapport between patients and clinicians.^31^

Currently, there is a lack of evidence on how the widespread uptake and ongoing use of remote consulting in UK outpatient services may have affected attendance rates overall and across patient groups. Rates of missed appointments may differ between in-person and remote appointments,^32^ particularly within first appointments as some patients may regard remote consultations as less suitable for first encounters than for follow-up care.^22,33^

The aim of this work was to explore the variation in rates of missed appointments by appointment modality (in-person or remote), at a large urban NHS Healthcare Trust in Northwest London, comparing first to follow-up appointments. As a secondary aim, we explored patient factors associated with non-attendance rates, comparing remote to in-person consultations.

## Methods

### Study design

We conducted a retrospective analysis of attendance of outpatient services at the Imperial College Healthcare NHS Trust (ICHT) in 2021, which includes five hospitals. All outpatient appointments which were booked to occur between 1st January 2021 and 31st December 2021 for adults (≥18 years at time of appointment) were extracted. COVID-19 lockdown restrictions were in place in the beginning of 2021 but were eased over the course of the year.^34^

### Data sources and data management

Anonymised electronic health records were accessed in the Northwest London Whole System Integrated Care (WSIC) database. This covers over 2.3 million patients, representing 95% of the Northwest London population.^35^ WSIC datasets are linked via a patient identifying key which enables integration of health records. Patient records used include secondary care outpatient data extracted based on the Secondary Uses Service data^36^ and patient sociodemographic and long-term conditions (LTCs) information compiled by the WSIC team from multiple WSIC datasets. Fully de-identified versions of WSIC data were analysed in the Discover-Now secure environment.^37^

### Study variables

The outcome variable considered was an appointment being missed. Outcomes were stratified by the type (*first* or *follow-up*) and mode (*in-person* or *remote*) an appointment was booked as, as shown in Box 1. Definitions for the mode, type and attendance status of an appointment are determined by NHS Digital as part of the processing cycle and data quality checks for commissioning datasets.^38^ A total of 4405 (2.3%) patients with missing information on age, gender, ethnicity or number of LTCs were excluded from analyses. An ‘Unknown’ category was retained for Index of Multiple Deprivation (IMD) Quintile as it was missing for a considerable number of patients. An overview of the predictors included in our analysis is provided in Box 1.

Box 1. Outcome and predictor variables.

**Table:** 

Variable	Description
*Outcome*	
Number of appointments missed per patient	A missed (DNA) appointment is defined within HES data as one which was not attended by the patient without having been cancelled in advance.
*Stratifying variables*	
Appointment type	First or follow-up. A first appointment was defined as the first appointment in an episode of care following from a referral, or the only appointment which took place for a particular patient excluding previous appointments which were booked but cancelled. All subsequent appointments within the same series are defined as follow-up appointments.
Appointment mode	Remote or in-person. Remote appointments as defined by NHS Digital for SUS outpatient data include telephone and telemedicine appointments. Telemedicine may include video or voice messaging services used to provide remote health assessments and therapeutic interventions.^38^
*Patient predictor variables*	
Age at appointment	The age in years of the patient at the time of the appointment, categorised into ten-year age-bands from 18 to 79, then an 80 + years group.
Gender	Male or female
Index of multiple deprivation (IMD) quintile	The deprivation level of the patients’ residential area, with a lower quintile indicating greater deprivation.
Ethnicity	Asian or Asian British, Black or Black British, Mixed, Not Stated, Other, White
Number of LTCs	Based on a set of 41 LTCs defined in the WSIC dataset as described previously.^39^

### Statistical analyses

The number of appointments were summarised by appointment type and patient characteristics (Box 1).

The per-patient number of missed appointments was analysed using negative binomial regression models stratified by appointment type and mode. A negative binomial distribution was used as the variance of count data was overdispersed compared to that expected under a Poisson distribution. Models were adjusted for patient predictor variables listed in Box 1, with an interaction term between age and gender. The model was offset by the total number of appointments (attended and missed) per patient to account for patients with multiple appointments in the period.^7^ Incident rate ratios (IRRs) and 95% confidence intervals (CI) were calculated. As a sensitivity analysis, to investigate potential explanations for differences in non-attendance rates between genders, models were re-run with the specialties of Obstetrics and Midwife Episode removed.

Marginal effects plots were produced for each model to summarise the role of predictors.^40,41^ Fitted values across each level of the predictor were calculated while holding categorical predictors constant at their proportion. Data were analysed in R version 4.2.1.^42^

### Ethics

Approvals and permissions to access the WSIC datasets for the purpose of service evaluation were granted by the Northwest London Sub-Data Research Access Group on 19th August 2021 (ID-138).

## Results

### Participant characteristics

There were 874,659 outpatient appointments for 189,882 patients across 47 specialties at ICHT between 1st January 2021 and 31st December 2021. Most patients were White (51.8%), aged under 60 years (63.7%), and female (61.4%) (Table 1). A breakdown of patient characteristics and appointment attendance by appointment type is given in Supplemental Tables S1 and S2.

**Table 1.** Patient characteristics, appointment attendance and appointment mode for outpatient appointments.

			In-person			Remote		
	Patients (*N* = 189,882)	Total appointments (*N* = 874,659)	Total (*N* = 644,388)	Missed (*N* = 65,132)	Missed rate (%)	Total (*N* = 230,271)	Missed (*N* = 25,166)	Missed rate (%)
**Age group**								
18 to 39	58,402 (30.8)	268,747 (30.7)	213,542 (33.1)	23,815 (36.6)	11.2	52,474 (22.8)	8,123 (32.3)	15.5
40 to 59	62,496 (32.9)	265,100 (30.3)	184,314 (28.6)	20,516 (31.5)	11.1	80,421 (34.9)	9,483 (37.7)	11.8
60 to 79	54,054 (28.5)	272,944 (31.2)	193,820 (30.1)	15,939 (24.5)	8.2	80,140 (34.8)	6,320 (25.1)	7.9
80+	14,930 (7.9)	67,868 (7.8)	52,712 (8.2)	4,862 (7.5)	9.2	17,236 (7.5)	1,240 (4.9)	7.2
**Gender**								0.0
Female	116,531 (61.4)	548,677 (62.7)	413,801 (64.2)	38,706 (59.4)	9.4	134,876 (58.6)	14,408 (57.3)	10.7
Male	73,351 (38.6)	325,982 (37.3)	230,587 (35.8)	26,426 (40.6)	11.5	95,395 (41.4)	10,758 (42.7)	11.3
**Ethnicity**								
White	98,372 (51.8)	444,429 (50.8)	323,846 (50.3)	28,810 (44.2)	8.9	120,583 (52.4)	11,796 (46.9)	9.8
Asian or Asian British	34,241 (18)	158,103 (18.1)	118,271 (18.4)	10,955 (16.8)	9.3	39,832 (17.3)	4,207 (16.7)	10.6
Black or Black British	24,726 (13)	124,806 (14.3)	93,222 (14.5)	12,596 (19.3)	13.5	31,584 (13.7)	4,161 (16.5)	13.2
Mixed	7,409 (3.9)	34,003 (3.9)	25,145 (3.9)	3,031 (4.7)	12.1	8,858 (3.8)	1,130 (4.5)	12.8
Not stated	5,997 (3.2)	25,418 (2.9)	18,523 (2.9)	2,052 (3.2)	11.1	6,895 (3)	895 (3.6)	13.0
Other ethnic groups	19,137 (10.1)	87,900 (10)	65,391 (10.1)	7,688 (11.8)	11.8	22,519 (9.8)	2,977 (11.8)	13.2
**IMD quintile**								
1 (most deprived)	45,284 (23.8)	223,421 (25.5)	165,502 (25.7)	19,658 (30.2)	11.9	57,919 (25.2)	7,352 (29.2)	12.7
2	57,485 (30.3)	263,644 (30.1)	194,304 (30.2)	20,149 (30.9)	10.4	69,340 (30.1)	7,600 (30.2)	11.0
3	44,274 (23.3)	200,418 (22.9)	147,479 (22.9)	13,347 (20.5)	9.1	52,939 (23)	5,425 (21.6)	10.2
4	24,236 (12.8)	102,414 (11.7)	74,534 (11.6)	6,034 (9.3)	8.1	27,880 (12.1)	2,592 (10.3)	9.3
5 (least deprived)	8,573 (4.5)	37,655 (4.3)	27,511 (4.3)	1,753 (2.7)	6.4	10,144 (4.4)	736 (2.9)	7.3
Unknown	10,030 (5.3)	47,107 (5.4)	35,058 (5.4)	4,191 (6.4)	12.0	12,049 (5.2)	1,461 (5.8)	12.1
**Number of LTCs**								
0	65,569 (34.5)	262,121 (30)	206,156 (32)	20,877 (32.1)	10.1	55,965 (24.3)	7489 (29.8)	13.4
1	45,259 (23.8)	205,123 (23.5)	147,898 (23)	14,800 (22.7)	10.0	57,225 (24.9)	6278 (24.9)	11.0
2	31,953 (16.8)	148,476 (17)	105,602 (16.4)	11,039 (16.9)	10.5	42,874 (18.6)	4481 (17.8)	10.5
3+	47,101 (24.8)	258,939 (29.6)	184,732 (28.7)	18,416 (28.3)	10.0	74,207 (32.2)	6918 (27.5)	9.3

Data are shown as *n* (% of *N*) unless specified as a rate.

### Remote appointments

Over a quarter (*n* = 230,271, 26.3%) of total appointments were booked as remote. A fifth (19.2%, *n* = 58,160) of 303,631 first appointments and 30.1% (*n* = 172,111) of 571,028 follow-up appointments were booked as remote. Remote appointment scheduling varied by specialty and appointment type (Supplemental Tables S3 and S4). Many patients (38.5%) had both a remote and in-person appointment booked in the study period.

### Missed appointments

The overall missed appointment rate was 10.3% (*n* = 90,298). Remote and in-person appointments had similar non-attendance rates overall, at 10.9% (*n* = 25,166) and 10.1% (*n* = 65,132), respectively.

The non-attendance rates for remote and in-person appointments differed over time and by appointment type (Supplemental Figure S1). Within first appointments, a total of 29,710 appointments were missed and remote appointments were more often missed throughout the year than in-person appointments (12.5 vs 9.2%, *p* < 0.0001). Within follow-up appointments, 60,588 appointments were missed and remote and in-person modalities had similar non-attendance rates (10.4 vs 10.7%, *p* = 0.001) (Supplemental Figure S1).

Non-attendance rates varied by specialty (Supplemental Table S3 and S4). Non-attendance rates were highest for Clinical Immunology and Allergy (16.3%) and lowest for Clinical Oncology, Medical Microbiology & Virology, and Radiology, all of which had a non-attendance rate of 1.4%. The specialties with the greatest number of missed appointments by volume were Midwife Episode (*n* = 9,348; 10.4% of total missed appointments) and Ophthalmology (*n* = 8,833; 9.8% of total missed appointments).

About 30% (*n* = 56,152) of patients accounted for all missed appointments. Of the 139,146 patients who had a first appointment, 12.1% missed one appointment and 3.9% of patients missed multiple first appointments across different episodes of care (Supplemental Figure S2). Of the 140,322 patients who had a follow-up appointment, 19.8% missed one appointment and 9.5% missed multiple (Supplemental Figure S2).

### Patient predictors of missed appointments by appointment modality

IRRs and 95% CI for each model can be found in Supplemental Tables S5 and S6.

#### Ethnicity

**Figure 1.**
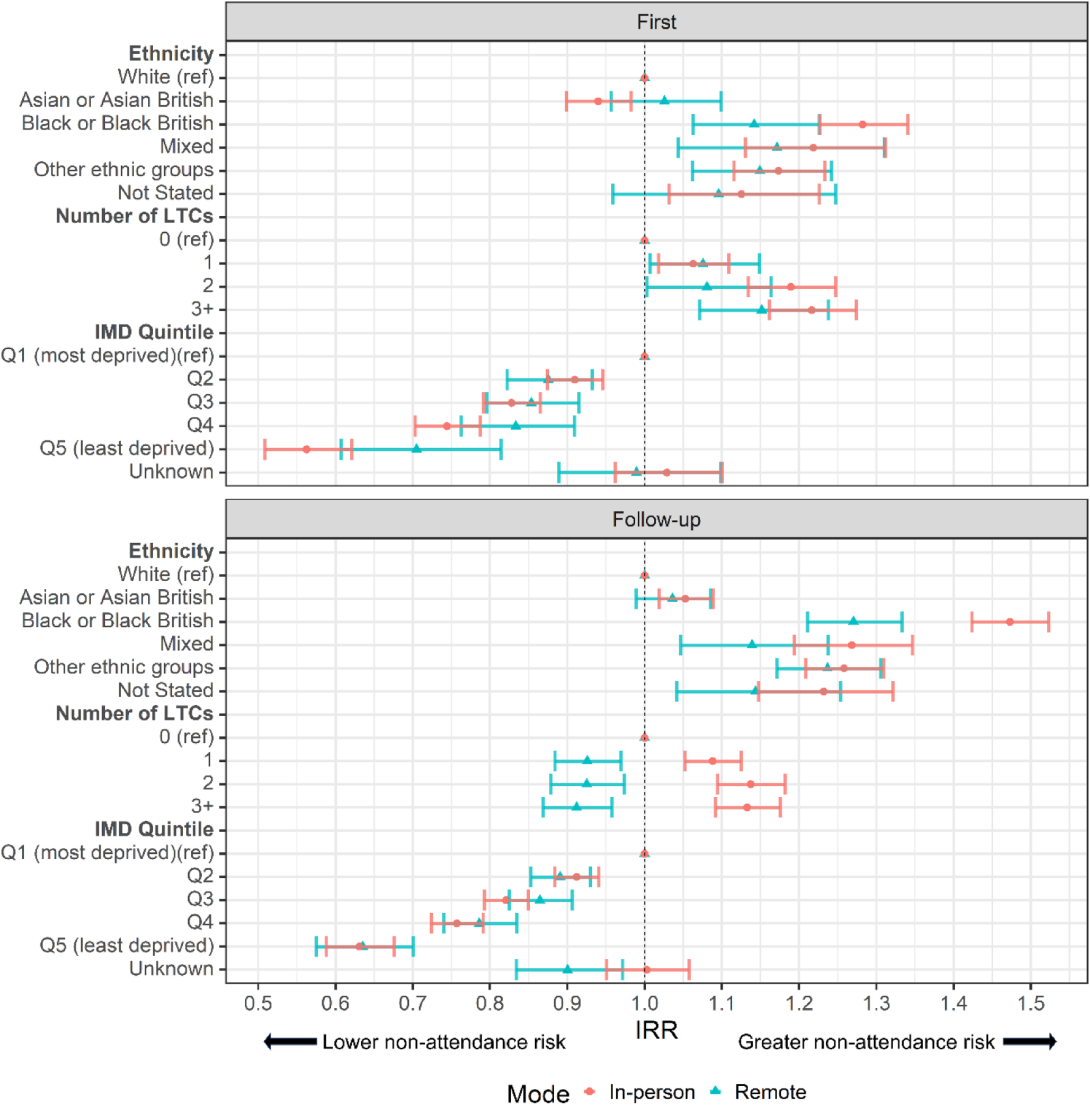
Incidence rate ratios (IRR) of missed appointments by mode for (A) first and (B) follow-up appointments. Ref = reference category. IRRs were derived from a negative binomial regression adjusted for patient age and gender (with an interaction), ethnicity, IMD quintile and number of LTCs, with an offset of total appointments per patient. Bars represent 95% CI.

Rates of missed appointments for remote and in-person first and follow-up appointments varied by patient ethnicity (Figure 1). For both in-person and remote first and follow-up appointments, patients of Black, Mixed and ‘Other’ ethnic groups had significantly higher non-attendance rates on average relative to White patients. Differences in non-attendance rates between some ethnic groups were more pronounced for follow-up compared to first appointments (Figure 1).

#### Number of LTCs

Within first appointments, patients with one or more LTCs had higher adjusted non-attendance rates relative to patients with no LTCs regardless of modality (Figure 1(A)). In-person follow-up appointments for patients with at least one LTC were more likely to be missed, follow-up appointments booked as remote for patients with at least one LTC were less likely to be missed (Figure 1(B)).

#### IMD quintile

Residence in areas of lower deprivation was associated with lower adjusted rates of missed appointments relative to the most deprived quintile, for all appointment types and modes (Figure 1).

#### Age and gender

**Figure 2.**
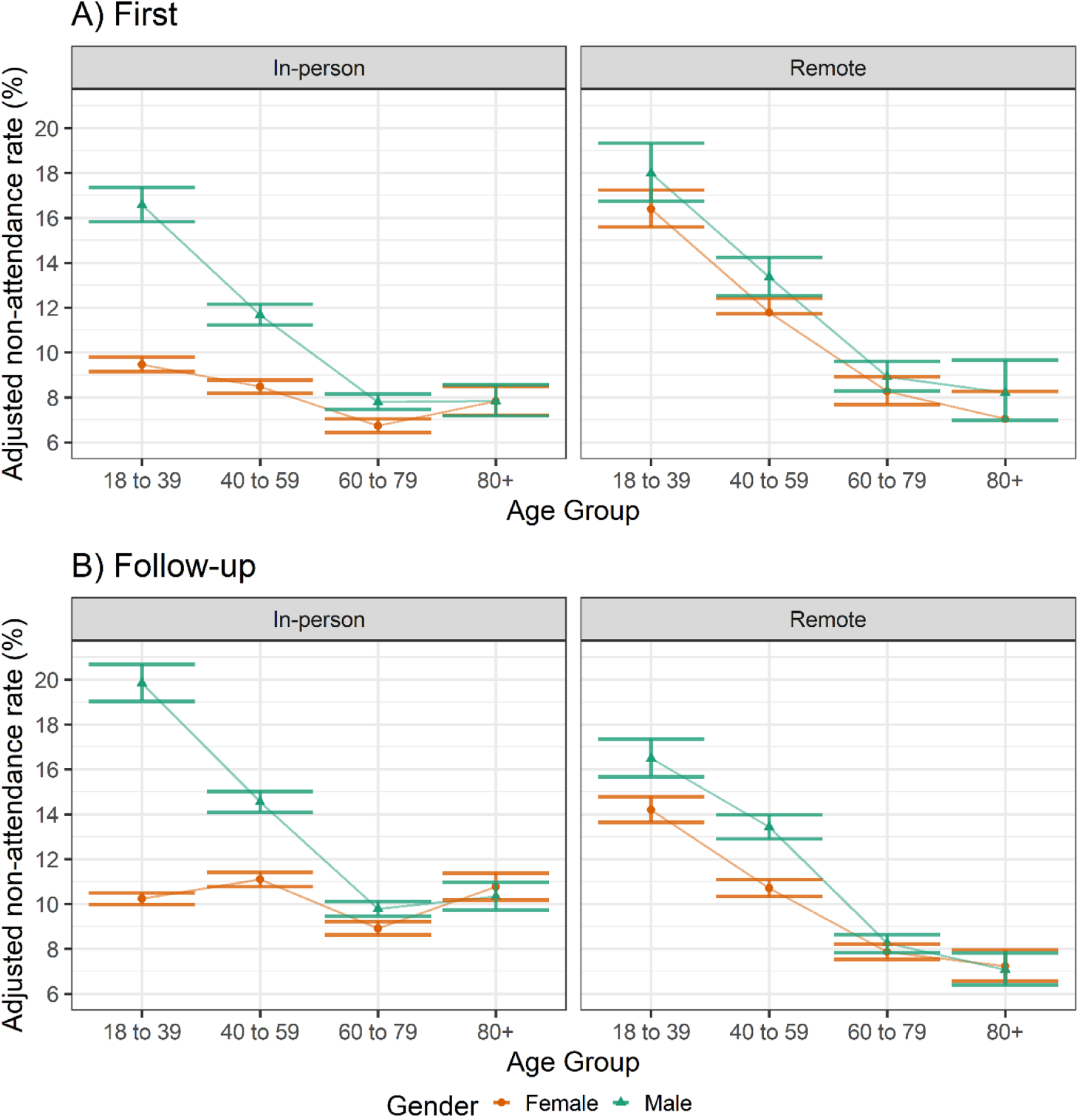
Adjusted non-attendance rates across age groups and genders for (A) first and (B) follow-up appointments. Derived from a negative binomial regression model adjusted for ethnicity, IMD quintile and number of LTCs, and offset for total appointments made per patient.

Non-attendance rates for first appointments decreased with increasing age for both genders regardless of modality, but to differing degrees between genders for in-person appointments (Figure 2). Female patients had lower non-attendance rates than male patients within in-person first appointments between the ages 18 to 79, particularly at younger age groups (Figure 2(A)).

Older patients were less likely to miss a follow-up appointment within in-person appointments for males, and within remote appointments for both genders. In-person follow-up appointments for females showed little difference in non-attendance rates across age groups (Figure 2(B)).

A sensitivity analysis removing maternity-related specialties from the analyses was conducted. The sensitivity analysis reduced the difference in non-attendance rates for in-person appointments between male and female patients in the age group 18 to 39 but to a greater degree within first appointments, resulting in a similar trend in non-attendance rates of in-person appointments with increased age in both genders (Supplemental Figure 3). The sensitivity analysis had little effect upon remote appointments as relatively few appointments for these specialties were booked to occur remotely (Supplemental Tables S3 and S4).

## Discussion

### Summary of key findings

Between 1st January 2021 and 31st December 2021, 10.3% of appointments at Imperial College Healthcare NHS Trust were missed, accounting for a total of 90,298 appointments. For first appointments in an episode of care, remote appointments were missed more frequently than in-person appointments (12.5 vs 9.2%, *p* < 0.0001). For follow-up appointments, the rates of missed appointments were similar overall between remote and in-person appointments (10.4 vs 10.7%, *p* = 0.001).

Socio-demographic differences in rates of missed appointments were largely similar regardless of whether the appointment was booked as in-person or remote, or if it was a first or follow-up appointment. This suggests no apparent widening of inequalities in attendance based on the modality of the appointment. However, our findings do indicate significant inequalities in rates of missed appointments overall: there were greater non-attendance rates for younger age groups, residents of more deprived areas, patients with LTCs (for in-person appointments only), and for people of Black, Mixed and ‘other’ ethnicities.

### Comparison with literature

We report a 10.3% non-attendance rate for the Trust in 2021. This estimate aligns with an analysis of outpatient data at ICHT, which documented a non-attendance rate of 11.2% for the period 2017 to 2018,^43^ a time pre-COVID-19 pandemic when remote consulting was less common at the Trust. However, this figure exceeds the 6.4% rate of missed appointments estimated by NHS England nationally for the year 2021/2022^8^ which could be attributed to the setting of the Trust in a relatively young and ethnically diverse population.^5^ Certain patients exhibit patterns of multiple missed appointments, often spanning across primary and secondary care, which likely reflects unmet or unaddressed healthcare needs.^7,44^ Our study demonstrated this phenomenon in the NWL population, as fewer than 30% of patients were responsible for all missed appointments at the Trust.

Remote first appointments were more frequently missed, but remote and in-person follow-up appointments had similar attendance, which may indicate a patient preference for initial appointments to be carried out in-person.^33^ The finding that appointment non-attendance was largely similar for remote, in-person, first, and follow-up appointments suggests that there are underlying reasons for non-attendance shared across appointment modes and types. Factors such as competing work or family commitments, patient forgetfulness and difficulties with appointment booking systems have been identified as reasons for non-attendance,^2,45–47^ and these issues are not specific to a particular mode of appointment delivery. Changes to appointment mode are also unlikely to address the impacts of negative experiences and distrust of health systems on attendance.^26^ Although we did not find evidence of remote appointments contributing to digital exclusion of at-risk groups, digital exclusion may still be occurring through other components of an increasingly digitised healthcare system. For example, patients with low digital literacy or English language skills may have difficulty using appointment management systems, resulting in misunderstandings about appointment details or an inability to reschedule or cancel an appointment.^47,48^

For follow-up appointments, having one or more LTC was associated with higher non-attendance for in-person appointments but lower non-attendance for remote appointments. COVID-19 may have motivated patients with certain LTCs to avoid in-person interactions where possible, resulting in non-attendance of in-person follow-up appointments. Travel considerations, severe illness, or the need to arrange support from a carer may also contribute to patients with LTCs being less able to attend in-person appointments.^49^ Remote appointments have reduced travel requirements and facilitate self-management capabilities which may improve outpatient attendance in patients with LTCs.^50^ That patients with LTCs were of greater risk of non-attendance for first appointments, regardless of modality, is concerning, given the association between poor attendance and negative health outcomes in these groups.^1,4–6^

The greater non-attendance in younger patients and residents of more deprived areas is consistent with previous data on missed appointments at the Trust^43^ and in other NHS secondary and tertiary care settings.^5^ Concerns that appointments conflict with work or childcare commitments would be less relevant to retired and more affluent populations and would contribute to fewer missed appointments in these groups. These results further support findings that populations associated with poorer health and more complex needs – such as those from deprived areas, ethnic minority groups, and patients with LTCs – have a higher risk of non-attendance.^32,51,52^ These health and sociodemographic risk factors for non-attendance often co-occur,^32^ potentially with a compounding effect upon non-attendance risk and consequent negative health outcomes.

Missing or withheld sociodemographic data may indicate a lack of engagement with health services that contributes to appointment non-attendance. The observation that patients who did not state their ethnicity were at greater non-attendance risk has previously been observed in ophthalmic outpatient appointments.^32^ Similarly, we observed patients with an unknown deprivation level as having comparable non-attendance risk to those resident in the most deprived areas. Missing deprivation data could indicate a lack of interaction with multiple health and social care organisations, as this information was sourced from multiple NHS datasets, including primary, secondary, community, emergency and tertiary care. These findings support the argument that missing data as an indicator of engagement patterns could be used to target preventative measures at low-engagement patients and mitigate associated negative health outcomes.^7,32^

An analysis of ICHT outpatient data from 2017 to 2018 found that the importance of demographic factors in predicting attendance varied by specialty.^43^ In our examination of in-person appointments, removing appointments for the specialties of Maternity and Obstetrics from the regression analyses resulted in higher average non-attendance rates for younger female patients. Some of the Trust-wide differences in non-attendance rates between genders were therefore driven by the differing healthcare needs of male and female patients. Further, first appointments showed patterns of missingness across ethnic groups and patients with different numbers of LTCs which were distinct to those seen within follow-up appointments. This may reflect differing utility of remote mediums of communication between initial and review encounters with patients.^13^ Together these findings highlight the importance of contextual factors in predicting appointment attendance.

### Strengths and limitations

To our knowledge, this is the first investigation of the use of remote as compared with in-person appointments in secondary care, achieved through exploration of a large dataset of secondary care appointments which provides near-comprehensive coverage of the Northwest London population. Through linkages to multiple NHS datasets, we were able to include patient demographic variables as confounders in our analyses.

However, the study has limitations. Data were from a single NHS Trust period and therefore these findings may not generalise to other NHS Trusts. In this context, remote appointments largely referred to telephone appointments. Factors which could have affected attendance might differ between video and telephone appointments, and between real-time and asynchronous delivery modes,^32^ however, we were unable to examine this aspect as the data did not distinguish between varieties of remote appointment delivery. Moreover, we lacked information regarding the extent to which patients or clinicians had a choice in the method of appointment delivery, or what motivated an appointment to be booked as a particular mode. Access to remote appointments is known to vary demographically due to factors such as age, disability status, income, education level and ethnicity.^27^ Bias likely arose from risk-stratification processes which aimed to offer remote consultations only where suitable to the needs and abilities of the patient.^13^

### Implications for health policy and practice

We found no influence of sociodemographic factors on attendance at remote as compared with in-person appointments. However, we established further evidence of inequalities in an individuals’ likelihood to miss healthcare appointments, with lower overall attendance rates for younger age groups, residents of more deprived areas, and for people of Black, Mixed and ‘Other’ ethnicities compared to those of White ethnicity. Policy makers and health providers should explore ways to identify individuals at risk of missing appointments, with a view to establishing interventions to mitigate this risk.

While we identified minimal difference in non-attendance rates of follow-up appointments based on appointment modality, first outpatient appointments were more frequently missed when booked as remote, compared to in-person. This may suggest patient preference for initial visits within an episode of care to occur in person. Healthcare providers making use of remote consultations as part of secondary care pathways should therefore exercise caution in the routine use of remote consultations for first outpatient appointments to reduce the risk of missed appointments.

### Implications for future research

This study examined secondary care data within a large, linked dataset. We identified differences between first and follow-up appointments both in the frequency of remote appointments and in patterns of missed appointments across patient groups. Further research could examine the factors contributing to the differing attendance rates and utility of remote care between first and follow-up appointments, particularly for patients with LTCs. Continued research into remote service delivery and factors associated with attendance is necessary to avoid the entrenching or exacerbation of existing health inequalities in appointment attendance and any resulting differences in health outcomes.

Many of the patient groups identified here to be of higher risk of outpatient appointment non-attendance are also at risk of missing appointments for other health services, including primary care.^44^ An understanding of patient journeys through multiple health and social care systems would be beneficial in identifying and addressing the factors contributing to missed appointments across multiple levels of care. This could be achieved through a mixed-methods approach involving qualitative exploration of reasons for non-attendance supported by linked primary and secondary care appointment data. Initially, we aimed to also explore primary care attendance, but coded data on consultation modality in primary care was not available. We have written elsewhere of the urgent need for improvements in coded primary care data.^53^ Linked datasets such as WSIC offer opportunities for effective service planning, implementation, and evaluation as well as for identifying individuals in need of tailored healthcare services, with the goal of improving health outcomes and healthcare system efficiency. However, their value is limited by data availability and quality; being routinely collected data, these datasets do not include patient experience or patient-reported outcomes. Future research should investigate the impact of in-person and remote consultations in other regions of London and beyond, using comprehensive primary care and secondary care data. Collaboration among researchers, policymakers, healthcare providers and practitioners is crucial to develop strategies for improving healthcare coding across diverse settings.

## Conclusions

Identification of methods to enhance efficiency and accessibility, as well as address the ‘wastage’ associated with missed appointments, is a priority for healthcare providers. Given the well-established links between missed appointments and health inequalities, it is essential to that new models of care aimed at improving efficiency and access neither exacerbate existing inequalities nor create new imbalances in care provision.

This study may provide reassurance to healthcare providers that a move towards remote outpatient consultation provision seems unlikely to have increased the risk of missed appointments due to new factors relating to consultation modalities. However, it also reinforces evidence of differences in missed appointments that may result from and exacerbate health inequalities for certain sociodemographic groups. This highlights the need for policymakers and healthcare providers to offer targeted support for improving accessibility and attendance. Furthermore, indications of a potential patient preference for in-person over remote consultations for first outpatient appointments is an important consideration for healthcare providers in designing and implementing new care pathways.

## Supplemental Material

sj-docx-1-jtt-10.1177_1357633X231216501 - Supplemental material for Attendance at remote versus in-person outpatient appointments in an NHS Trust Supplemental material, sj-docx-1-jtt-10.1177_1357633X231216501 for Attendance at remote versus in-person outpatient appointments in an NHS Trust by Gabriele Kerr, Geva Greenfield, Benedict Hayhoe, Fiona Gaughran, Kristoffer Halvorsrud, Mariana Pinto da Costa, Nirandeep Rehill, Rosalind Raine, Azeem Majeed, Ceire Costelloe, Ana Luisa Neves, and Thomas Beaney in Journal of Telemedicine and Telecare
